# Restoration of miR-23a expression by chidamide sensitizes CML cells to imatinib treatment with concomitant downregulation of CRYAB

**DOI:** 10.1080/21655979.2022.2056322

**Published:** 2022-03-25

**Authors:** Xunxun Zhu, Jingru Zhang, Yanping Sun, Yan Wang, Qian Liu, Peng Li, Shuang Yu, Na Liu, Jingjing Ye, Daoxin Ma, Chunyan Ji

**Affiliations:** a Department of Hematology, Qilu Hospital, Cheeloo College of Medicine, Shandong University, Jinan, shandong, China; b Department of Hematology, Tengzhou Central People’s Hospital, Tengzhou, Shandong, China; c Department of Hematology, Taian Central Hospital, Taian, Shandong, China

**Keywords:** Chronic myeloid leukemia, imatinib mesylate, miR-23a, CRYAB, chidamide

## Abstract

MicroRNAs (miRNAs) are involved in various processes from the initiation and development of cancers, including chronic myeloid leukemia (CML). In this report, we aimed to investigate the roles of miR-23a in the regulation of imatinib mesylate (IM) sensitivity in CML cells and the possible mechanisms involved in this process. We demonstrated that the expression of miR-23a was markedly low in bone marrow mononuclear cells from patients in whom IM treatment had failed and imatinib-resistant K562/G01 cells when compared to patients with optimal responses and imatinib-sensitive K562 cells, respectively. Overexpression of miR-23a was shown to induce apoptosis of K562/G01 cells and sensitize these cells to imatinib treatment. With the aid of bioinformatics analysis, we revealed that CRYAB could be a potential downstream effector of miR-23a, contributing to miR-23a-mediated IM resistance. We also observed that the expression of CRYAB was inversely correlated with miR-23a expression in CML cell lines and patient samples. Importantly, chidamide upregulated miR-23a expression and reversed the IM resistance of CML cells. Together, these findings strongly suggest that miR-23a acts as a tumor suppressor by downregulating CRYAB expression. Restoration of miR-23a by chidamide may therefore have a therapeutic effect in controlling the sensitivity of CML cells to imatinib.

## Graphical abstract

**Figure:**
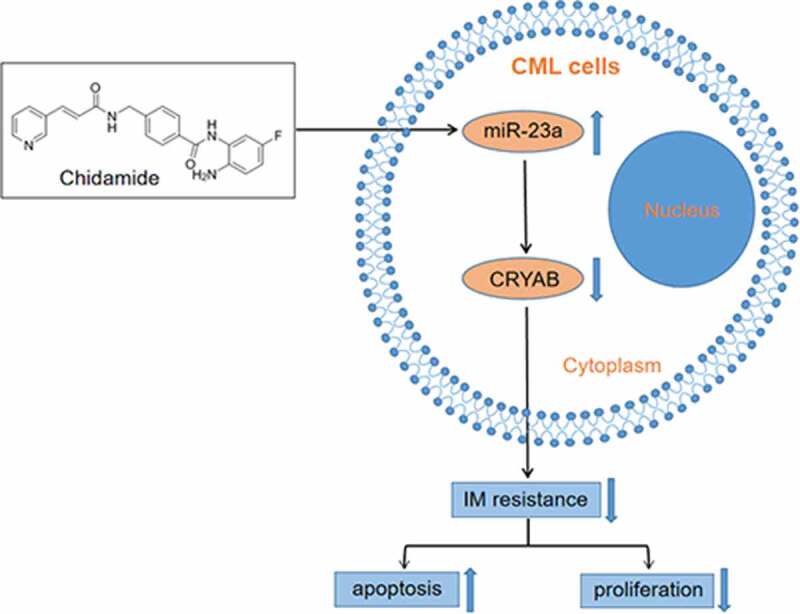

## Introduction

Chronic myeloid leukemia (CML) is a hematopoietic malignancy defined by the BCR-ABL fusion protein encoded by the Philadelphia chromosome (Ph)[1]. Through activating various signaling pathways, BCR-ABL causes cell proliferation and inhibits apoptosis [2,3]. On account of the wide application of tyrosine kinase inhibitors (TKIs), including the most well-known TKI imatinib mesylate (IM), the outcome in patients with CML has significantly improved[4]. However, drug resistance occurs in approximately 15–20% of patients, resulting in a major obstacle in IM treatment[5]. Hence, exploring the mechanisms underlying IM resistance and identifying new effective markers to distinguish which patients are likely to progress are indispensable research directions.

MicroRNAs (miRNAs) are small RNAs with 19–24 nucleotides[6]. Aberrant and absent expression of miRNAs have been reported in various tumors [7,8]. MiRNAs are considered to be oncogenes and tumor suppressors. Therefore, it might mediate chemoresistance as well as pathogenesis [9,10]. Abnormally expressed miRNAs have been observed in patients with IM-resistant CML. Specifically, miR-101, a tumor suppressor, sensitizes CML cells to IM by downregulating JAK2 expression[11]. Upregulated expression of miR-202-5p, the downstream target gene of STAT5, inhibits cell apoptosis and promotes resistance to IM[12]. The identification emphasizes the need to consider miRNA-based treatment methods in addition to traditional therapies[13]. Recently, the therapeutic relevance of miR-23a was revealed by a growing body of evidence. In solid tumors, miR-23a can mediate resistance to erlotinib[14], 5-fluorouracil[15], and cisplatin[16]. Nevertheless, the functions and underlying mechanisms of miR-23a in IM resistance in CML have not been documented.

This study explored the roles of miR-23a in mediating sensitivity to IM in CML patients. Here, we aimed to investigate whether the upregulation of miR-23a expression in CML cells enhanced sensitivity to IM via CRYAB inhibition. Moreover, overexpression of miR-23a by chidamide treatment alleviated the resistance of CML cells to IM. Thus, our study forecasted the potential of miR-23a as a molecular target for treating IM-resistant CML.

## Material and methods

### Ethical statement

The Ethics Committee of Qilu Hospital (affiliated with Cheeloo College of Medicine, Shandong University) approved the study (approval number, KYLL-2014(KS)-033; Date, 25 February 2014), and informed consent was obtained from all individuals according to the Declaration of Helsinki.

The ethical approvement was provided in supplementary document.

### Patient samples

Seventy bone marrow (BM) specimens were collected from CML patients (44 males and 26 females, 19–75 years old) who received IM treatment at Qilu Hospital from May 2012 to April 2017. The newly diagnosed patients were treated with 400 mg IM daily. Of the 70 samples, 25 were obtained from patients with optimal responses (OR), and 45 were obtained from patients in whom IM treatment had failed (TF).Twenty-six normal control (NC) samples were obtained from healthy individuals. In addition, matched BM samples were obtained from 7 CML patients with optimal responses in the chronic phase (CP) and in the blastic phase (BP) in whom IM treatment had failed. Data on all CML patients were collected just one year after IM treatment. The patient characteristics, white blood cell count, and BCR-ABL levels are shown in Table 1. The diagnosis and classification of CML were established according to the recommendations of the National Comprehensive Cancer Network (NCCN) Clinical Practice Guidelines in Oncology, Chronic Myeloid Leukemia. Ficoll density gradient centrifugation was used to extract mononuclear cells (MNCs) from BM. The isolated MNCs were stored at −80°C.

**Table 1.** Clinical characteristics of patients

Characteristics	Optimal responses (n = 45)	Treatment failure (n = 25)
Age at diagnoses (years)		
media	45	42
range	19–75	23–70
sex		
male	29	19
female	16	6
BCR-ABL(international standard) (%)	0.31 ± 0.64	11.16 ± 17.38
White blood cells (10^9^/L)	11.35 ± 5.86	20.73 ± 9.42
The time of IM treatment (days)	183.17 ± 32.23	199.10 ± 56.34
BCR-ABL mutation rate (%)	2.2%	19.23%

### Cell culture and reagents

The cell lines used in this study, including the drug-sensitive K562 and the drug-resistant variant K562/G01, were obtained from the Institute of Hematology & Blood Diseases Hospital in China (Tianjin, China). Both cell lines were cultured under standard conditions with complete RPMI-1640 medium (HyClone, Logan, UT, USA) supplemented with 10% fetal bovine serum (TBD, Haoyang, China) and 1% penicillin and streptomycin (Invitrogen, Carlsbad, CA). To maintain the drug-resistant phenotype, the medium of the K562/G01 cells was supplemented with IM at a final concentration of 0.5 μmol/L (μM) until two weeks before the assay. IM came from Sigma-Aldrich (St. Louis, MO, USA). Chidamide was provided by Shenzhen Chipscreen Biosciences, Ltd. (Shenzhen, China).

### Quantitative real-time PCR

Total RNA was extracted with TRIzol reagent (Invitrogen, USA). miR-23a expression was measured using a TaqMan MicroRNA assay (Ambion Inc., Austin, TX, USA) and was normalized to U6 expression. cDNA was synthesized by a pair of special stem–loop primers, and another pair of primer/probe sets was used to conduct qPCR. The sequences of the miR-23a primers were F: 5’-GCCCATCACATTGCCAGG-3’ and R: 5’-GTGCAGGGTCCGAGGT-3’. To measure CRYAB (alpha B-crystallin) expression, reverse transcription of cDNA from total RNA was conducted with an RTase cDNA Synthesis Kit (Takara, Dalian, China). Quantitative real-time PCR was implemented utilizing SYBR Green PCR Master Mix (Toyobo, Osaka, Japan) with an Applied Biosystem 7900HT system (ABI, Foster City, CA, USA) using the ∆∆C_t_ method. The CRYAB primer sequences were F: 5’-AACGGCCTGGGTGGATAGAAG-3’ and R: 5’-CAGTACTCACTGAGCTGCTCTT-3’. The primer sequences are shown in Table S1. The CRYAB expression results were normalized to GAPDH expression. All reactions, including those with nontemplate controls, were performed in triplicate.

### Cell transfection with miRNAs and siRNAs

The synthetic miR-23a mimic, miR-23a inhibitor and CRYAB siRNA were composited by RiboBio (Guangzhou RiboBio Co., Ltd.). The cell lines were transfected with Lipofectamine 2000 reagent (Invitrogen, Carlsbad, CA).

### Chemosensitivity assay

A CCK-8 Kit (Beyotime, China, JS) was used to study drug sensitivity. Cells were counted and the concentration was adjusted to 5 × 10^4^, and then seeded in 96-well plates. K562/G01 cells were cultured with IM at graded concentrations of 0, 1, 2, 4, 8, 16, and 32 μmol/L. For K562 cells, IM was used at concentrations of 0, 0.1, 0.2, 0.4, and 0.8 μmol/L. The concentrations of chidamide were 0, 1.2, 2.4, and 4.8 μmol/L. After 48 h of treatment, CCK-8 solution was added to each well. The absorbance at 450 nm was measured by an automated microplate spectrophotometer (Thermo, USA). The IC50 value, that is, the concentration of one drug needed to inhibit cell growth by 50%, was calculated after correcting for background absorbance.

### Flow cytometric analysis of apoptosis

Cells were preincubated with IM (0.1 μmol/L for K562 cells and 3 μmol/L for K562/G01 cells) for 48 h in 12-well plates. A total of 2 × 10^5^ resuspended cells were dyed with 5 μL Annexin V/FITC, followed by 1 μL PI. Finally, the fluorescence of at least 1 × 10^5^ cells was examined by a FACSCalibur flow cytometer (Becton Dickinson, CA, USA).

### Gene chip analysis

Total RNA was extracted after miR-23a overexpression. A NanoDrop ND-1000 was used to detect RNA quantity and quality. Sample labeling and array hybridization were accomplished following the Agilent One-Color Microarray-Based Gene Expression Analysis protocol (Agilent Technology). Microarray image analysis and standard enrichment computation analysis were accomplished by KangChen Biotech (Shanghai, China).

### Western blotting

K562/G01 cells were collected, centrifuged, and lysed in radioimmune precipitation assay (RIPA) buffer supplemented with protease and phosphatase inhibitors. A BCA protein assay was performed to assess the protein concentration. Forty micrograms of protein per sample was fractionated by SDS–PAGE and then transferred to nitrocellulose membranes (Millipore, Billerica, MA). Afterward, the membranes were incubated with the following specific antibodies: anti-CRYAB (Abcam, Cambridge, MA, USA) and anti-β-actin (Cell Signaling Technology, New England BioLabs Inc., USA). The protein bands were visualized by the LI-COR infrared imaging system (Lincoln, NE). Full-length images of the blots and gels are presented in Figure S1.

### Statistical analysis

All data represent at least three independent experiments and are denoted as the mean ± SD. Two-tailed Student’s t test and one-way analysis of variance were performed for statistical comparison. Differences in miR-23a expression between clinical groups were determined with the Mann–Whitney test. All analyses were accomplished with SPSS software (version 17.0). Asterisks indicate that the values are significantly different (* *p* < 0.05; ** *p* < 0.01; *** *p* < 0.001).

## Results

In this study, the roles of miR-23a in mediating IM sensitivity will be explored, based on samples from CML patients, IM-sensitive and IM-resistant CML cells. We found that upregulation of miR-23a in CML cells enhanced the sensitivity to IM via CRYAB inhibition. Overexpression of miR-23a by chidamide treatment could alleviate the IM resistance of CML cells. Thus, our study will provide new insights into miR-23a as a potential molecular target in treating IM-resistant CML.

### MiR-23a expression was significantly downregulated in patients in whom IM treatment had failed and in IM-resistant cells

**Figure 1.**
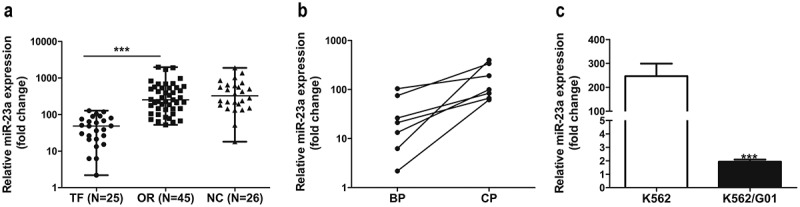
Expression levels of miR-23a in CML patient specimens and cell lines. (a) qRT–PCR results of miRNA-23a expression levels in CML patient specimens with treatment failure (TF, n = 25), optimal responses (OR, n = 45) and normal controls (NC, n = 26). Data are presented as the median and interquartile range. ***p < 0.001. (b) qRT–PCR results of miRNA-23a expression in the BP group of patients in whom IM treatment failed and the matched CP group with optimal responses. (c) Expression levels of miRNA-23a in two CML cell lines. The miRNA-23a levels in K562/G01 cells are displayed as fold changes in K562 cells. ***p < 0.001.

Initially, to investigate whether miR-23a impacts chemosensitivity to IM, we analyzed clinical samples. Figure 1(a) shows that miR-23a levels were significantly reduced in patients in whom IM treatment had failed compared to those with optimal responses and normal controls. Moreover, the data in the BP group of patients in whom IM treatment had failed was markedly lower than that in the parental CP group with optimal responses (Figure 1(b)). In agreement with the clinical results, miR-23a was reduced in K562/G01 cells compared to K562 cells (Figure 1(c)). These results indicate that miR-23a downregulation correlates with IM resistance in CML.

### miR-23a affects the chemosensitivity of CML cells to IM

**Figure 2.**
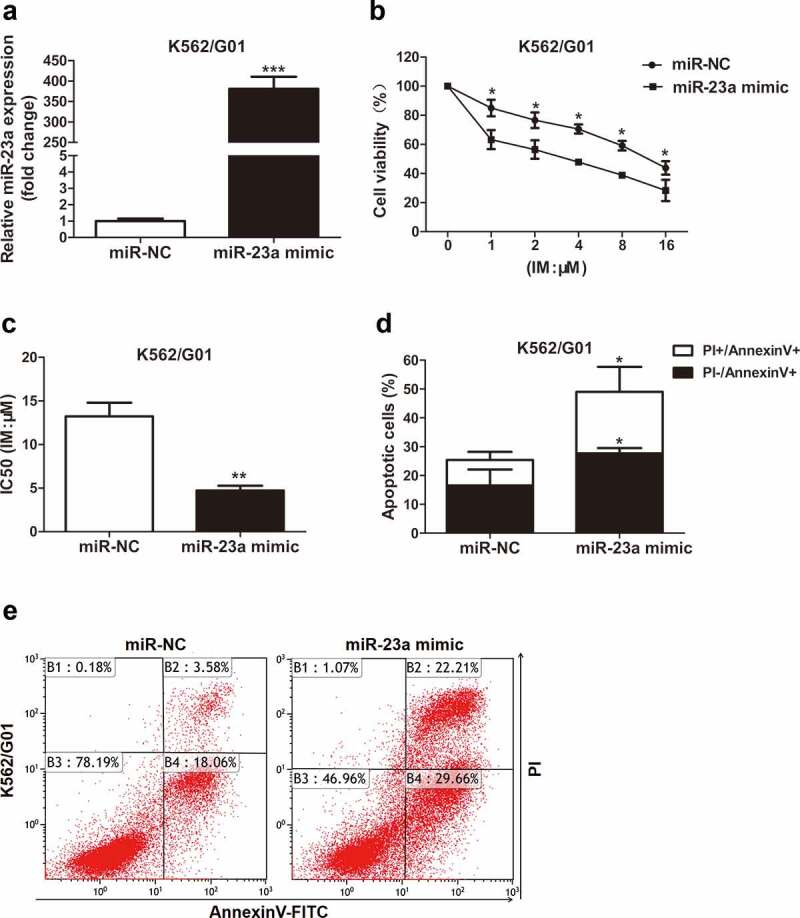
Sensitivity to IM after miR-23a overexpression in K562/G01 cells. (a) K562/G01 cells were transfected with an overexpression construct (miR-23a mimic) or negative control (miR-NC). The expression levels of miR-23a were detected by qRT–PCR and are displayed as fold changes in the NC-transfected cells. ***p < 0.001. A CCK-8 assay was conducted to assess the cell viability (b) and IC50 values (c) of K562/G01 cells after transfection and IM treatment. *p < 0.05, **p < 0.01. (d and e) Flow cytometry analysis of apoptosis in K562/G01 cells cultured with IM for 48 h after transfection. *p < 0.05.

**Figure 3.**
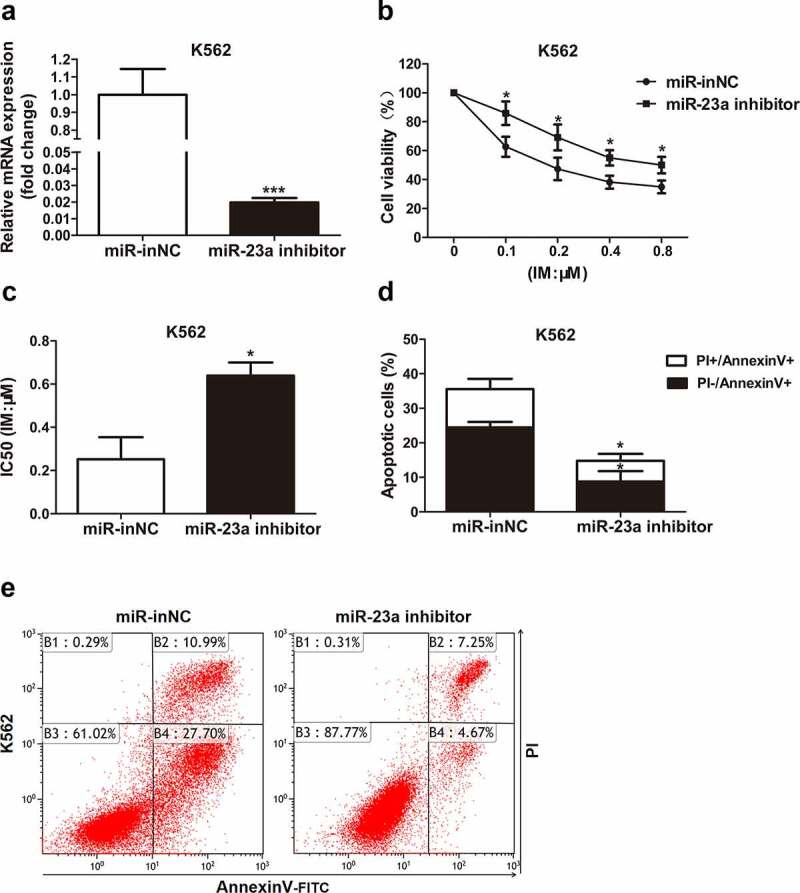
Sensitivity to IM after miR-23a downregulation in K562 cells. (a) K562 cells were transfected with the miR-23a inhibitor or negative control (miR-inNC). The expression levels of miR-23a were detected by qRT–PCR and are displayed as fold changes in the inNC-transfected cells. ***p < 0.001. A CCK-8 assay was conducted to assess the cell viability (b) and IC50 values (c) after transfection and IM treatment. *p < 0.05. (d and e) Flow cytometry analysis of apoptosis in K562 cells cultured with IM for 48 h after transfection. *p < 0.05.

To further delineate whether it affects the sensitivity to IM, miR-23a was overexpressed in K562/G01 cells. The chemosensitivity of these cells to IM was then examined with CCK-8 assays, as measured by cell viability. Upregulated miR-23a expression was shown by qRT–PCR analysis (Figure 2(a)). As shown in Figure 2(b–c), compared to negative control, the cell viability was reduced in K562/G01 cells after transfection, demonstrating that miR-23a overexpression exhibited greatly enhanced sensitivity to IM. Flow cytometry analysis revealed that miR-23a upregulation obviously enhanced the proportion of apoptotic K562/G01 cells (Figure 2(d–e)). These results could be verified by the downregulation of miR-23a. Figure 3(a)shows the effectiveness of the miR-23a inhibitor. Indeed, suppression of miR-23a expression resulted in decreased sensitivity to IM in K562 cells (Figure 3(b–c)). Furthermore, IM-induced apoptosis was decreased in K562 cells when miR-23a was downregulated (Figure 3(d-e)). These data indicate that overexpression of miR-23a affects chemoresistance to IM in CML cells.

### CRYAB is regulated by miR-23a

**Figure 4.**
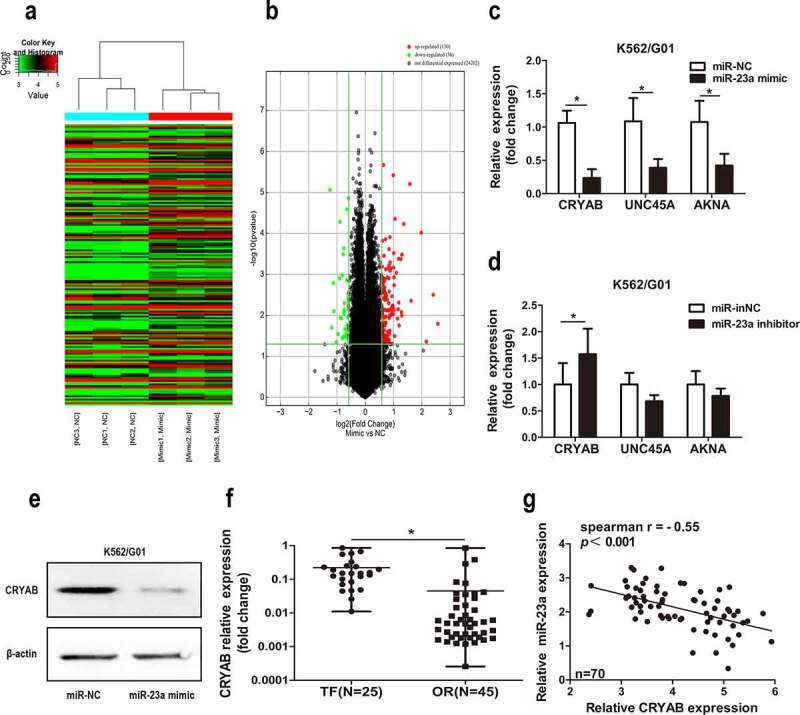
Expression of CRYAB regulated by miR-23a. Heatmap (a) and volcano plot (b) of all the genes expressed differentially in K562/G01 cells after miR-23a overexpression. ‘Red’ denotes upregulated expression; ‘green’ denotes downregulated expression; ‘black’ denotes nondifferential expression. (c) Expression of CRYAB, UNC45A, and AKNA in K562/G01 cells was detected under the premise of miR-23a overexpression. Data are expressed as the mean ± SD. *p < 0.05. (d) Experiments were repeated after miR-23a was downregulated. Data are expressed as the mean ± SD. *p < 0.05. (e) WB analysis was conducted to verify the downregulation of CRYAB protein expression in cells with miR-23a overexpression. (f) qRT–PCR demonstrating CRYAB expression in CML patients in whom IM treatment had failed (TF, n = 25) and in those with optimal responses (OR, n = 45). The data are presented as the median and interquartile range. *p < 0.05. (g) The correlation between CRYAB and miR-23a was detected in CML patient specimens. Correlation coefficients were calculated by Spearman-Rho.

Next, to determine the mechanism behind miR-23a-mediated chemotherapeutic resistance to CML, we employed gene chip analysis to screen for potential target genes (Figure 4(a-b)). A detailed list of the gene expression results is presented in Table S2. Subsequently, we performed a literature analysis of the top 30 genes, screening for a link not only to drug resistance but also to cancer. Three related genes (CRYAB, UNC45A, and AKNA) met all these requirements. Next, we correlated miR-23a with these genes and revealed the regulatory effect of CRYAB by miR-23a only. As shown in Figure 4(c), the expression levels of CRYAB, UNC45A and AKNA were decreased when miR-23a was overexpressed. Conversely, only the level of CRYAB was increased with miR-23a knockdown (Figure 4(d)). This result was also confirmed by Western blot analysis (Figure 4(e)). Moreover, increased mRNA levels of CRYAB were observed in patients in whom IM treatment had failed compared with patients with optimal responses (Figure 4(f)). Figure 4(g) shows the inverse correlation between CRYAB and miR-23a in CML clinical samples. The above data indicate that CRYAB is a potential downstream effector of miR-23a and might contribute to miR-23a-induced IM resistance in CML.

### CRYAB affects IM sensitivity

**Figure 5.**
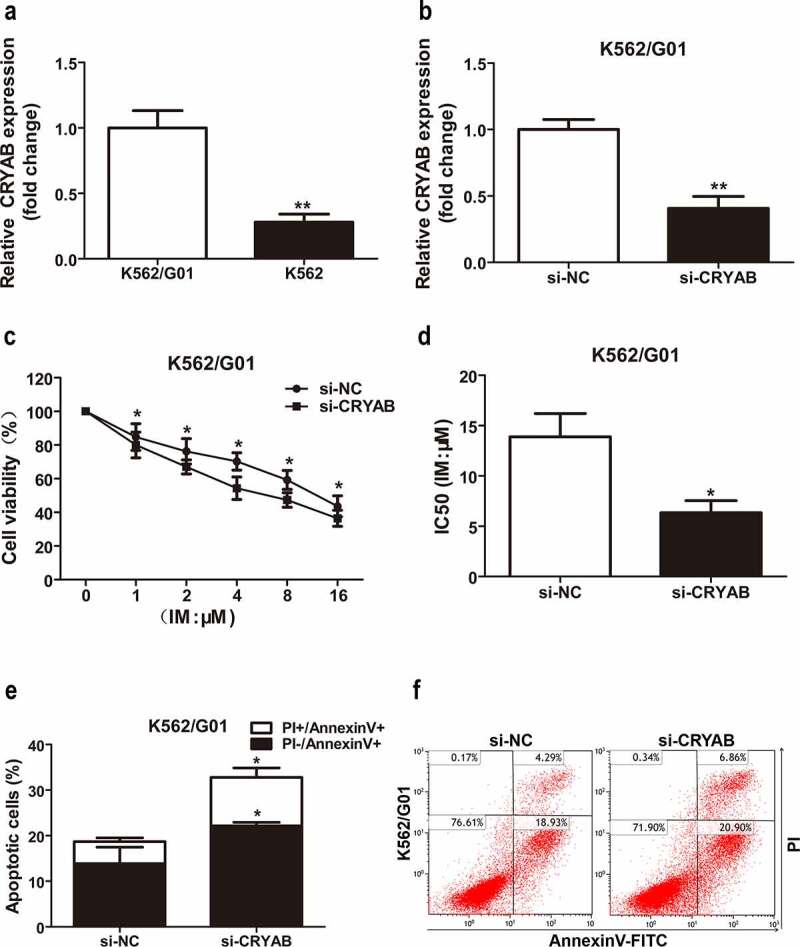
Sensitivity to IM after CRYAB knockdown. (a) Expression levels of CRYAB in two CML cell lines. The CRYAB levels in K562/G01 cells are displayed as fold changes in K562 cells. **p < 0.01. (b) CRYAB expression after transfection with CRYAB siRNA (si-CRYAB) or negative control (si-NC) in K562/G01 cells. Data are expressed as the mean ± SD. **p < 0.01. (c) A CCK-8 assay was conducted to assess the viability of K562/G01 cells incubated with IM with a knockdown of CRYAB. *p < 0.05. (d) The IC50 values of the conditions mentioned above are shown. *p < 0.05. (e and f) Flow cytometry analysis of apoptosis in cells cultured with IM for 48 h after transfection with si-CRYAB. *p < 0.05.

Since overexpression of miR-23a leads to chemosensitivity to IM in CML cells, we speculated that CRYAB downregulation could also cause chemosensitivity to IM. To verify this hypothesis, we first measured the CRYAB expression status in CML cell lines. The results showed higher expression levels of CRYAB in IM-resistant K562/G01 cells than in IM-sensitive K562 cells (Figure 5(a)). Next, we silenced CRYAB expression in K562/G01 cells with specific siRNAs, and the effectiveness of silencing CRYAB expression is shown in Figure 5(b). CRYAB downregulation significantly inhibited K562/G01 cell proliferation and increased cell sensitivity to IM, as shown by CCK-8 analysis (Figure 5(c–d)). To further confirm these results, we analyzed IM-induced cell apoptosis with flow cytometry analysis. The analyses revealed that the cell apoptosis proportion was substantially increased in the CRYAB siRNA-transfected cells (Figure 5(e–f)). Indeed, inhibition of CRYAB expression may contribute to miR-23a-induced cell apoptosis and IM sensitivity in CML.

### Chidamide upregulated miR-23a expression and reversed the IM resistance of CML cells

**Figure 6.**
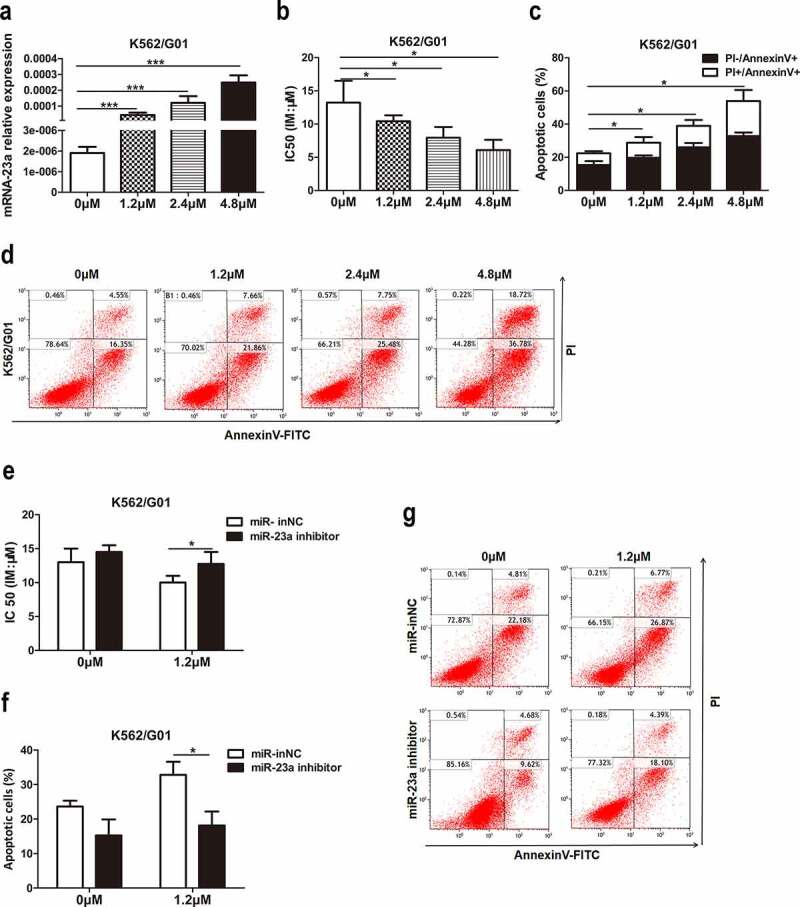
Effects of chidamide on miR-23a expression and IM resistance in CML cells. (a) qRT–PCR results of miR-23a expression in K562/G01 cells treated with serial dilutions of chidamide. ***p < 0.001. (b) CCK-8 assays in chidamide-treated K562/G01 cells after cultured with IM. The IC50 values are shown. *p < 0.05. (c and d) Flow cytometry analysis of apoptosis in chidamide-treated K562/G01 cells after cultured with IM. *p < 0.05. The expression of miR-23a was downregulated by an inhibitor, and the experiments mentioned above were repeated. The IC50 values (e) and flow cytometry analysis of apoptosis (f and g) are shown. *p < 0.05.

Previous studies suggested that miR-23a could be epigenetically activated by a DNA methylation inhibitor[17]. However, the effect of histone acetylation modification on miR-23a and IM resistance is still not understood, leading us to evaluate the effect of the histone deacetylase inhibitor (HDACi) chidamide on miR-23a-mediated IM resistance. First, we treated K562/G01 cells with chidamide at increasing concentrations for 24 h. The data demonstrated an increasing trend of miR-23a expression after treatment with chidamide (Figure 6(a)). We were interested in whether chidamide could mediate resistance to IM. For this purpose, we cultured these cells with IM and performed CCK-8 and flow cytometry analyses. Indeed, the chemosensitivity to IM (Figure 6(b)) and IM-induced apoptosis proportion (Figure 6(c–d)) were gradually increased, which suggests that chidamide could reduce K562/G01 cell resistance to IM. Subsequently, to explore whether miR-23a plays a role in the chidamide-induced increase in sensitivity to IM, we cocultured miR-23a inhibitor-transfected K562/G01 cells with chidamide and IM. As shown in Figure 6(e–g), downregulation of miR-23a expression attenuated the chidamide-mediated inhibition of cell proliferation and increased apoptosis to a certain degree. These data provide evidence that chidamide could enhance chemosensitivity to IM partially through upregulating miR-23a expression.

## Discussion

Although the emergence of TKIs, such as imatinib, has revolutionized the treatment of CML, chemoresistance remains an obstacle to successful treatment. Identifying molecular targets that affect chemoresistance and using them to establish novel therapies, if successful, has great potential to improve patient survival. Increasing evidence shows that BCR-ABL-independent factors influence TKI chemoresistance, including CML stem cells[18], genomic instability, and abnormal molecular signaling pathways[19]. However, these perspectives do not fully elucidate the BCR-ABL-independent mechanism underlying IM resistance.

Among the constantly growing list of candidate genes, miRNAs have become a hot topic in the study of chemorefractory disease. Here, we studied the function of miR-23a in chemoresistance to IM, which is a classic treatment protocol for CML. We found that decreased miR-23a expression reduces sensitivity to IM in CML cell lines and is associated with treatment failure in CML specimens, suggesting its pivotal role in drug resistance and disease progression. Human miR-23a is located in the cluster miR-23a-27a-24-2[20], and its function is controversial and correlates with the cell type, target gene and stage of cell differentiation. Hu X et al. reported that miR-23a/b promotes the progression of gastric cancer through PDCD4[21]. Jiang Y et al. found that the proliferation of hepatocellular carcinoma cells is promoted by the miR-23a-Stat5A-Akt signaling pathway[22]. Consistent with solid tumor studies, miR-23a has been reported to mediate cytarabine resistance in AML cells by targeting the TOP2B gene and to correlate with the relapsed/refractory stage[23]. However, other authors have validated that miR-23a is involved in the inhibition of tumor cell proliferation. Wang G et al. confirmed that miR-23a inhibits osteosarcoma growth by targeting SATB1[24]. Ma M et al. reported that miR-23a-3p inhibits the growth and progression of mucosal melanoma[25]. Ganesan S et al. showed that the expression of miR-23a was increased in acute promyelocytic leukemia (APL) cells compared to other acute leukemia cells[26]. This may be one of the explanations for the sensitivity of APL cells to arsenic trioxide and anthracycline. Overall, these results establish miR-23a as a potentially important target for the treatment of chemorefractory disease.

To explore the mechanisms underlying miR-23a downregulation-mediated IM resistance, we predicted the target genes of miR-23a and validated CRYAB as its downstream gene using a microarray and miR-23a mimic. CRYAB, a small heat-shock protein, is a recognized antiapoptotic protein[27]. Studies have shown that CRYAB is involved in cancer migration, chemoresistance, and poor prognosis [28,29]. Nevertheless, the antiapoptotic effect of CRAYB in CML has rarely been investigated. In this study, we demonstrated that miR-23a downregulated CRYAB expression at the mRNA and protein levels. Further knockdown of CRYAB expression, simulating the role of miR-23a overexpression, led to the reversal of chemoresistance to IM. Therefore, it is rational to assume that miR-23a increases CML sensitivity to IM by directly or indirectly targeting CRYAB. Notably, each miRNA regulates the expression of multiple targets[30], and the degree of regulation depends on distinct clonal architectures[31]. Thus, the low expression of miR-23a in CML cells may lead to specific dysregulated gene expression profiles, including not only CRYAB but also many other genes. Nevertheless, our study has certain limitations, and future studies describing the approach of miR-23a in regulating CRYAB or other potential target genes are necessary.

In addition, the development of new drugs that focus on chemoresistant CML cells is crucial. In some cancer cells, miR-23a expression can be regulated by drugs. As reported by Lu B et al., andrographolide can enhance the expression of miRNA-23 in liver cancer cells[32]. Wang N et al. found that the expression of miR-23a can be regulated by berberine[33]. Here, we found that chidamide, a novel HDACi independently developed in China, may induce apoptosis and increase CML sensitivity to IM by regulating miR-23a expression. Consistently, a recent study validated that HDACi suppress the proliferation and tumorigenicity of drug-resistant stem cells in CML via regulation of miR-196a expression [34]. However, the detailed underlying mechanisms by which chidamide regulates TKI resistance and the mechanisms of crosstalk between chidamide and the miR-23a/CRYAB pathway in CML are still unknown and need to be explored in future research.

## Conclusion

In conclusion, we combined clinical and experimental studies to determine the role and clinical significance of miR-23a in IM resistance of CML. We speculate that the expression of CRYAB in CML patients can predict the response to TKIs and is an early warning indicator for the progression of CML disease. Furthermore, we highlighted the essential role of chidamide as an emerging component of CML treatment.

## Supplementary Material

Supplemental Material
