# Supplementary material for: Restoration of miR-23a expression by chidamide sensitizes CML cells to imatinib treatment with concomitant downregulation of CRYAB
Source: Bioengineered. 2022 Mar 25;13(4):8881–92. doi: 10.1080/21655979.2022.2056322 (PMC9162009; doi:10.1080/21655979.2022.2056322)
Supplement: Supplemental Material [file KBIE_A_2056322_SM7890.pdf]

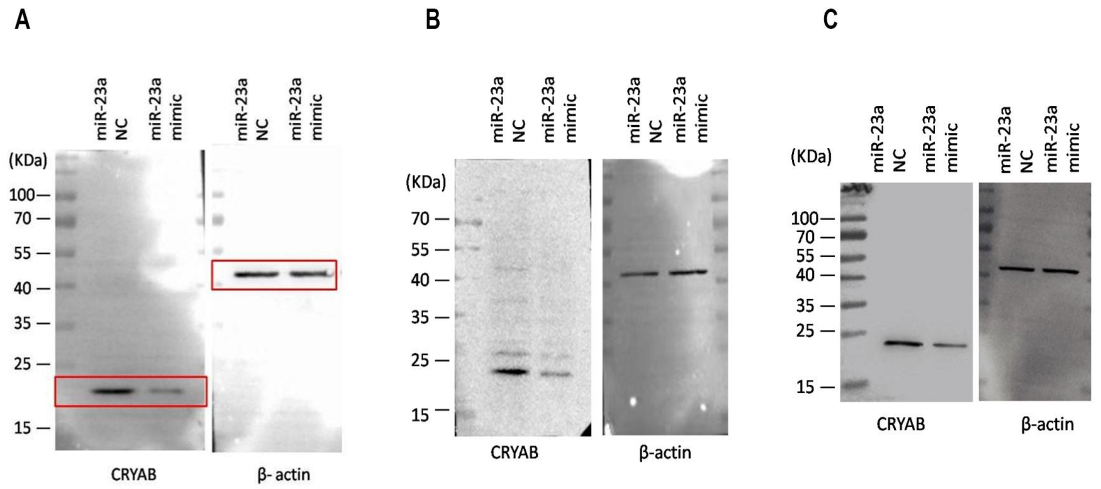

Figure S1. (A) Full-length images of blots and gels presented in Figure 4e. Rectangles indicate the regions used in the figure. (B-C) The experiment was repeated three times.

Table S1. List of primers used in this study

Name	Forward primer	Reverse primer
miR-23a mimic	5’-AUCACAUUGCCAGGGAUUUCC-3’	5’-AAAUCCCUGGCAAUGUGAUUU-3’
miR-23a NC	5’-UUCUCCGAACGUGUCACGUTT-3’	5’-ACGUGACACGUUCGGAGAATT-3’
miR-23a Inhibitor	5’-GGAAAUCCCUGGCAAUGUGAU-3’	
miR-23a Inhibitor NC	5’-CAGUACUUUUGUGUAGUACAA-3’	
CRYAB	5’-AACGGCCTGGGTGGATAGAAG-3’	5’-CAGTACTCACTGAGCTGCTCTT-3’
UNC45A	5’-TTGCGAGTCACCGAGTTTCC-3’	5’-CTGACCTTVTCTGGATGCCC-3’
AKNA	5’-AGGTCCCCTGAAACTGGGT-3’	5’-ACCTGGGCCACTTCATCTTC-3’
GAPDH	5’-UGACCUCAACUACAUGGUUTT-3’	5’-AACCAUGUAGUUGAGGUCATT-3’
SiRNA-CRYAB	5’-GCUGGUUUGACACUGGACUTT-3’	5’-AGUCCAGUGUCAAACCAGCTT-3’
SiRNA-NC	5’-UUCUCCGAACGUGUCACGUTT-3’	5’-ACGUGACACGUUCGGAGAATT-3’
miR-23a	5’-GCCCATCACATTGCCAGG-3’	5’-GTGCAGGGTCCGAGGT-3’

Table S2. Candidate genes decreased significantly in K562/G01 cells after transfected with miR-23a mimic.

ProbeName	Fold Change	Regulation	GeneSymbol	Description
A_24_P331779	1.5575297	down	RSPH4A	Homo sapiens radial spoke head 4 homolog A
A_32_P331052	1.5217162	down	RBBP8NL	Homo sapiens RBBP8 N-terminal like (RBBP8NL), mRNA
A_23_P215913	1.8818085	down	CLU	Homo sapiens clusterin (CLU), transcript variant 1, mRNA
A_23_P126349	2.1562199	down	BARHL2	Homo sapiens BarH-like homeobox 2 (BARHL2), mRNA
A_23_P371145	1.5140382	down	ADPRHL1	Homo sapiens ADP-ribosylhydrolase like 1, transcript variant 1, mRNA
A_33_P3245439	1.5049747	down	CD40	Homo sapiens CD40 molecule, TNF receptor superfamily member 5, mRNA
A_33_P3549091	1.7477739	down	LOC100131347	Homo sapiens RAD52 motif 1 pseudogene , non-coding RNA
A_33_P3676515	1.7167975	down	SSR4P1	Homo sapiens signal sequence receptor, delta pseudogene 1， non-coding RNA
A_33_P3215640	1.5064945	down	PI16	Homo sapiens peptidase inhibitor 16 (PI16), transcript variant 1, mRNA
A_23_P33664	1.9507795	down	ELSPBP1	Homo sapiens epididymal sperm binding protein 1 (ELSPBP1), mRNA
A_23_P1523	2.2670929	down	RHOD	Homo sapiens ras homolog family member D (RHOD), mRNA
A_32_P146659	1.6368952	down	ATP6V0E2-AS1	Homo sapiens ATP6V0E2 antisense RNA 1， long non-coding RNA
A_23_P428887	1.6701633	down	KLHL34	Homo sapiens kelch-like family member 34 (KLHL34), mRNA
A_33_P3233560	1.5189255	down	UROS	uroporphyrinogen III synthase
A_23_P129332	1.7504647	down	PKD1L2	Homo sapiens polycystic kidney disease 1-like 2, transcript variant 3, mRNA
A_24_P365349	1.568168	down	CACNG7	Homo sapiens calcium channel, voltage-dependent, gamma subunit 7 , mRNA
A_33_P3297853	2.1782652	down	AKNA	Homo sapiens AT-hook transcription factor (AKNA), mRNA
A_33_P3316493	1.506118	down	WDR91	Homo sapiens WD repeat domain 91 (WDR91), mRNA
A_23_P28139	1.6182312	down	SCTR	Homo sapiens secretin receptor (SCTR), mRNA
A_24_P206776	2.3753049	down	CRYAB	Homo sapiens crystallin, alpha B (CRYAB), mRNA
A_33_P3367731	1.6938696	down	SLC24A2	Homo sapiens solute carrier family 24, member 2
A_23_P62752	1.672272	down	NPPB	Homo sapiens natriuretic peptide B (NPPB)
A_23_P431410	1.776934	down	RBMS1	Homo sapiens RNA binding motif, single stranded interacting protein 1ant 1
A_23_P120056	1.8554772	down	RTKN	Homo sapiens rhotekin (RTKN)
A_33_P3374394	2.0317225	down	UNC45A	Homo sapiens unc-45 homolog A
A_23_P348524	1.6741271	down	C20orf201	Homo sapiens chromosome 20 open reading frame 201
A_23_P372988	1.7187113	down	PRR22	Homo sapiens proline rich 22 (PRR22), mRNA
A_23_P351535	1.519162	down	DBIL5P	Homo sapiens diazepam binding inhibitor-like 5, pseudogene (DBIL5P)
A_23_P45324	1.8344228	down	TMEM35	Homo sapiens transmembrane protein 35 (TMEM35), mRNA
A_33_P3229477	1.5679959	down	MPP7	Homo sapiens membrane protein, palmitoylated 7, mRNA

山东大学齐鲁医院科研伦理委员会

批　准　书

批准号：KYLL-2014(KS)-033

项目名称： FoxM1 调控 DNA 损伤修复在慢性髓性白血病伊马替尼耐药中的作用及机制研究

项目负责人： 章静茹　**职称：** 医师　**联系电话：** 18560087016

负责研究单位： 山东大学齐鲁医院

合作研究单位：

研究起止时间： 2015.01-2017.12

拟申报项目类别及资助金额（或在研项目资金来源及金额）：

青年科学基金，25 万元

评审意见：

研究项目

“ FoxM1 调控 DNA 损伤修复在慢性髓性白血病伊马替尼耐药中的作用及机制研究”

经伦理委员会审查：

研究者的资格、经验符合试验要求；研究方案符合科学性和伦理原则的要求；获得知情同意的方法适当；受试者可能遭受的风险程度与研究预期的受益相比合适。

同意开展该项目的研究。

山东大学齐鲁医院科研伦理委员会

主任委员： 陈阳印鉴

2014 年　2 月 25 日

山东大学齐鲁医院科研伦理委员会 ETHICS COMMITTEE ON SCIENTIFIC RESEARCH OF SHANDONG UNIVERSITY QILU HOSPITAL 3701027061615
